# Supplementary material for: Heat adaptation of phage T7 under an extended genetic code
Source: Virus Evol. 2021 Dec 1;7(2):veab100. doi: 10.1093/ve/veab100 (PMC8923235; doi:10.1093/ve/veab100)
Supplement: veab100_Supp [file veab100_supp.zip › Sequencing_Analysis.zip!/Sequencing_Analysis/A2/calibration/0.unique_coverage.pdf]

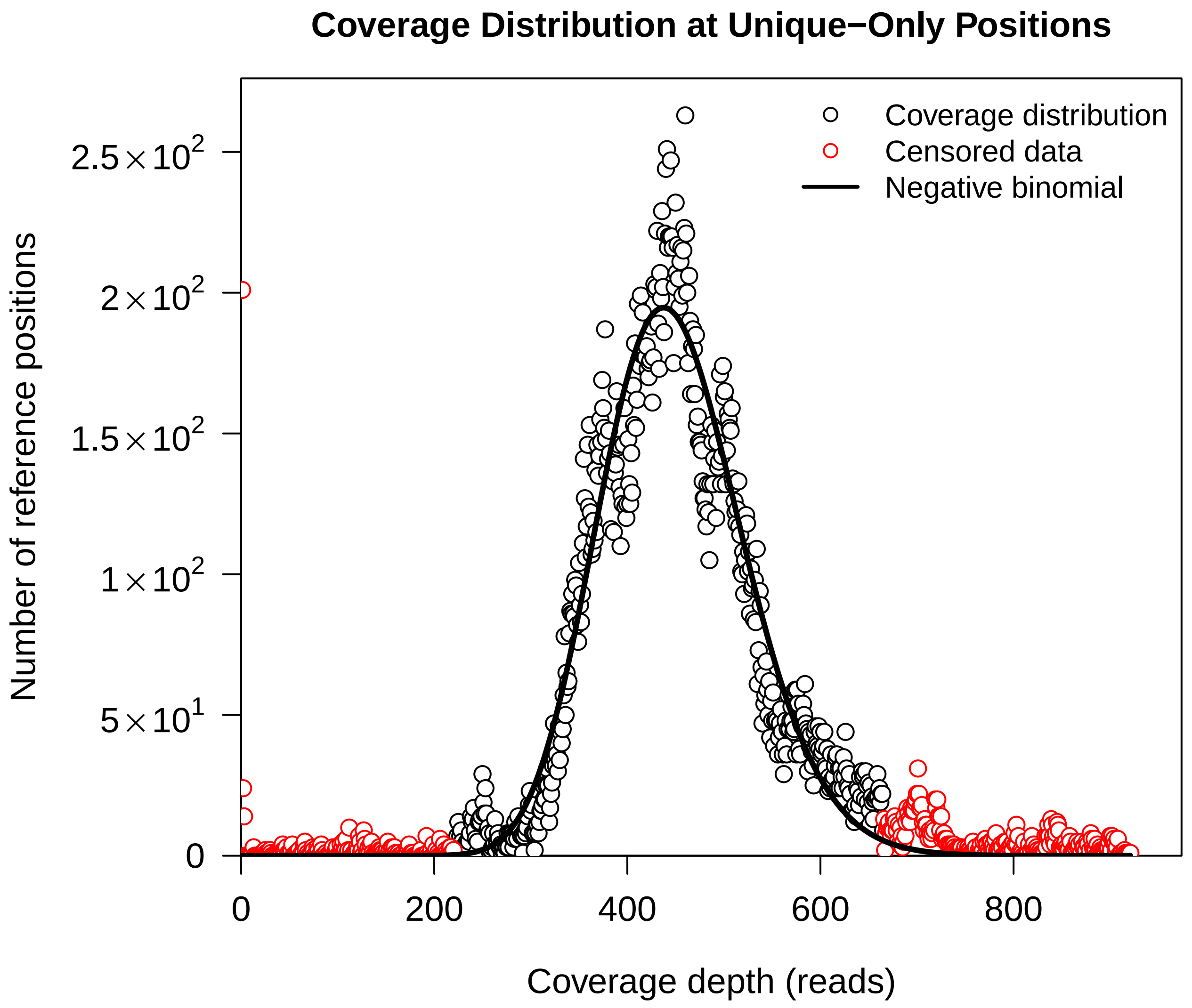

Coverage Distribution at Unique−Only Positions
Coverage distribution
Censored data
Negative binomial
Number of reference positions
Coverage depth (reads)
0
5×10^1
1×10^2
1.5×10^2
2×10^2
2.5×10^2
0
200
400
600
800
